# Mechanisms underpinning the onset of seed coat impermeability and dormancy-break in *Astragalus adsurgens*

**DOI:** 10.1038/s41598-019-46158-z

**Published:** 2019-07-04

**Authors:** Ganesh K. Jaganathan, Jiajin Li, Matthew Biddick, Kang Han, Danping Song, Yashu Yang, Yingying Han, Baolin Liu

**Affiliations:** 1 Institute of Biothermal Technology, University of Shanghai for Science and Technology, Shanghai, 200093 China; 2 School of Biological Sciences, Victoria University of Wellington, PO Box 600, Wellington, 6012 New Zealand

**Keywords:** Plant ecology, Ecology

## Abstract

Impermeable seed coats, i.e. physical dormancy (PY) influence the germination ecology of plants from 18 angiosperm families. *Astragalus adsurgens* (Fabaceae; Papilinoidaae) is a perennial plant widespread in temperate regions that is thought to produce both permeable and impermeable seeds. Why seeds vary in the permeability of their coat, in addition to the mechanisms by which impermeable seeds break dormancy, are not completely understood. However, seeds are often consumed by herbivores; a phenomenon that might facilitate the germination of impermeable seeds. Here, we tested whether: (1) moisture content plays a significant role in the onset of seed coat impermeability (and therefore PY) at similar ranges reported for species from tropical ecosystems; and (2) the presence of impermeable coats offer any benefits for seed survival when consumed by animals. We tested these hypotheses using *A*. *adsurgens* seeds collected from Inner Mongolia, China. Freshly collected seeds with a moisture content of 9.7% were permeable to water and therefore not physically dormant. However, seeds became impermeable when dried below a threshold of 6.5% moisture content. Treating impermeable seeds with hydrochloric acid effectively broke dormancy. Scanning electron microscope (SEM) revealed that HCl treated seeds had a narrow opening in the hilum and extra-hilar regions, through which water entered. Seeds with impermeable coats survived significantly better than permeable seeds when consumed by cows. Irrespective of coat permeability, most seeds were egested between 12 and 24 h. In seeds that maintained dormancy after gut passage, this was broken by additional acid scarification. Overall results suggest that: (1) seed coat impermeability is induced by reduced moisture content; (2) imbibition primarily occurs at the hilum and extra-hilar region; and (3) impermeable seeds may benefit from endozoochory.

## Introduction

*Astragalus adsurgens* (Fabaceae: Papilionoideae) is a perennial plant inhabiting arid regions of China, as well as temperate North America, Siberia, Mongolia, and rarely in Japan^1,2^. It grows wild along the Yellow River valley in central and eastern China^3^ and Tibetan grasslands^4^. In Inner Mangolian grasslands of China, *A*. *adsurgens* is abundant and often consumed by grazing ungulates. Japanese populations of *A*. *adsurgens* are reported to possess impermeable coats, i.e. physical dormancy (PY) due to the presence of palisade layer^2^; although dormancy can be broken by treatment with sulphuric acid. Contrastingly, Tobe and Gao^5^ conducted extensive germination experiments on Chinese populations of *A*. *adsurgens*, and seeds germinated between 66 and 93% at a range of temperatures between 5 and 35 °C, suggesting seeds were not dormant. Thus, the question of whether *A*. *adsurgens* seeds have an impermeable seed coat remains controversial.

Prior studies suggest that a species with impermeable seeds only become dormant when seed moisture content reaches a specific threshold^6–13^. If seed moisture content remains above this threshold during shedding, seeds remain permeable to water and germinate at a range of temperatures. Whereas, if seeds are dried below this threshold, seeds become impermeable to water. The transition from permeable to impermeable is characterized by numerous changes in the seed coat, for example, the closing of the micropyle or chalaza at the terminal stages of maturation^14^. Once impermeability is induced, seeds require dormancy-breaking cues that open specialized structures known as ‘water-gaps’, through which water enters and hydrates the embryo^15^.

The drying of seeds depends considerably on the environmental conditions experienced during maturation. Accordingly, the ratio of permeable to impermeable seeds produced varies between years^16^. Indeed, populations of the same species inhabiting different locations vary in their ratio of permeable to impermeable seeds produced^17^. Though PY occurs globally, it is more prevalent in the dry tropical regions^18^, and less common in wet temperate regions^19^. However, climate change is exerting more pressure on plant species^20^, with climate projection models predicting temperate ecosystem to become warmer and drier in the near future^21^. Furthermore, seeds that are shed at higher moisture content might be dried to levels that could onset impermeability when they persist in the soil, i.e. secondary dormancy, a feature where seeds are dispersed as non-dormant from mother plant and they become dormant after shedding It is therefore crucial to understanding the mechanisms underpinning the onset of PY in temperate species.

Physical dormancy is broken when the specialized structures present in the seed coat forms a small opening often referred to as ‘water-gap’, through which water enters into the seed. Seasonal temperatures cause diurnal fluctuations that open water-gap structures^15,22–24^. Water-gaps have been identified in 17 out of 19 families with PY^15,25^. However, the morphological structures acting as water-gaps vary taxonomically. For instance, species from the three subfamilies of Fabaceae: Mimosoideae^26,27^, Papilionoideae^28–33^ and Caesalpinioideae^14,22,34–36^ imbibe water through the lens. However, in *Sophora alopecuroides* (Papilionoideae)^37^ and *S*. *tomentosa*^38^, water enters primarily through the hilar slit, with the lens serving as secondary opening. The occurrence of more than one water-gap structure is termed as ‘water-gap complex’^13,25^. More detailed studies in some Fabaceae species have also revealed that structures other than lens and hilar slit, e.g. pleurogram could act as a water-gap^39^.

Physical dormancy can also be broken by gut passage^40,41^. Unlike most dormancy breaking cues, passage through the hydrochloric acid of the gut erodes impermeable seed layers^42^. Although this erosion could occur in the structures acting as water-gap, there is evidence that the whole seeds could have cracks which facilitate the entry of water^40^. Thus, successful germination of these seeds depends principally -amongst other factors- on the duration seeds spend inside gut: too long time in the acid environment leads to damage of embryo, too short preserve impermeable nature of the coat^43^. However, several factors control how long seeds spend in an animal’s gut, including seed size and host gut size and diet. Whether seed coat impermeability is a decisive factor affecting seed viability during gut passage remains unknown.

Here, we asked following questions about the germination ecology of *A*. *adsurgens*: (1) do seeds maturing in Inner Mongolia, China have impermeable seed coats, thus PY? (2) If so, does moisture content control the onset of seed impermeability? (3) what structures act as water-gap? (4) what is the effect of HCl scarification on dormancy break? and (5) how does consumption by animals affect seed germination?

## Materials and Methods

### Seed collection site and procedure

**Figure 1:**
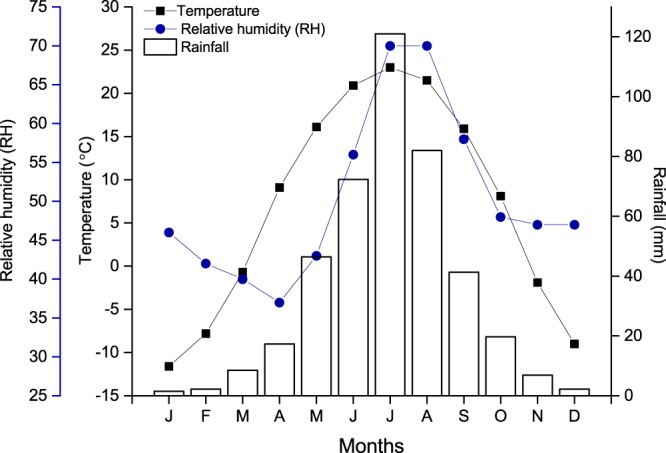
Monthly average temperature, relative humidity and rainfall recorded in the study site between 1986 and 2016.

Fully mature seeds of *A*. *adsurgens* were collected in grasslands of Inner Mongolia, China (41°17′10″N, 116°21′07″E) in mid-August 2017. Altitude ranges from 480–800 m.a.s.l. and the climate is cold-humid. Monthly average temperatures, relative humidity, and rainfall of the seed collection site over the past 30 years are presented in Fig. 1. The study site is dominated by species belonging to Poaceae, Cyperaceae, Ranunculaceae, Polygonaceae and Fabaceae.

Seeds were collected from at least 10 plants within two to three days after shedding and sent to University of Shanghai for Science and Technology, Shanghai, China within three days by air-freight. Seeds were stored in air-tight polythene bags after collection until received in the laboratory. Immediately after arrival in the laboratory, seeds were cleaned for debris and stored in air-tight glass bottles at 20–25 °C until used in the experiments. All experiments began within one week of collection.

### Seed weight and moisture content

The average weight of seeds was determined by weighing 10 replicates of 1000 randomly selected seeds in a standard digital balance. Moisture content was calculated by drying an aliquot of seeds at 103 °C for 17 h and determining the fresh and dry weight^44^. Three replicate of 2 g of seeds (approximately 2000 seeds) were used for moisture content determination throughout the study. Moisture content is expressed on a fresh weight basis.

### Imbibition and drying

Three replicates of 100 seeds were weighted in a digital balance (to the nearest 0.001 g) placed on a moist Whatman No.1filter paper in 90-mm Petri dishes at room temperature (20–22 °C). Seeds were reweighed at 2 h intervals for the first 8 h and then at every 8 h until 24 h. Percentage increase in seed mass was calculated using the formula derived by Baskin *et al*.^45^.

Batches of fresh seeds were dried above silica gel (1 seeds:20 silica gel, by weight) for 10, 20 and 30 and 60 h in air-tight containers. After drying to various time periods, the moisture content of the seeds was determined using three replicate of 1000 seeds following the method described above and a subset of 300 seeds each in three replicates of 100 seeds were subjected to an imbibition test to determine the ability of seeds to absorb water. Seed weight was determined every 2 h until first 8 h and after that every 12 h until 96 h. Number of seeds absorbing water was recorded.

### Seed germination

Four replicates of 100 seeds each were placed in Petri dishes on filter paper moistened with distilled water and incubated at the following temperatures: 5/10, 5/15, 10/15, 15/20, 20/25, 25/30, 30/35 °C. Seeds were incubated at 12/12 h photoperiod with approx. 40 μm m^−2^ s^−1^, 400–700 nm, cool white fluorescent light coincided with the warm phase in conditions with temperature fluctuations. In addition, seeds were also incubated under complete darkness. Seeds received darkness treatment were wrapped with aluminum foil and were exposed to green light during germination scoring, to avoid direct light exposure. Germination was scored every day for three weeks or until all the seeds had germinated with the aid of a magnifying lens. Germination was determined by visual confirmation of emergence of the radicle.

### Effect of HCl on dormancy break

Hydrochloric acid present in the intestine of consumers can break dormancy, by eroding the seed coat and creating spaces for the water to enter into seeds. To understand more on the effects of HCl on *A*. *adsurgens*, permeable and impermeable (by drying for 60 hours) seeds were treated with HCl (50% concentration) for 15, 30, 45 or 90 min and placed on moistened filter paper in Petri dishes to determine germination at 15/20 °C with a 12 h photoperiod. Three replicates of 100 seeds each were used.

### Seed coat features

To document structural changes that occur following acid treatment, eight seeds each treated with HCl for 15, 30, 45 or 90 min were mounted on aluminum SEM stubs and coated with gold alloy using a Technics Hummer VI sputter coater. These stubs containing seeds were then scanned in a FEI Quanta 450 field emission scanning electron microscope, and micrographs were compared with the impermeable seeds, that is seeds that had been dried for 60 hours and did not absorb water during imbibition test.

### Seed feeding trials

For the feeding trials, 1800 permeable and impermeable seeds each were fed to three healthy cows (six cows in total: three for permeable seeds and three for impermeable seeds) with an age of 5–8 years. These cows were given normal diet and water during the experimental period. Dung samples were collected for up to 32 h. For the purpose of this experiment, dung samples collected at different times were grouped as follows: 0–6, 6–12, 12–18, 18–24 and 24–32 h. Few impermeable seeds were egested after 32 h, but none of the seeds were intact and germinated when placed on a moist substrate, and thus were excluded from the analysis. Immediately after collection, dung samples were gently washed with water above cotton-woven towels and seeds were separated with the aid of a magnifying lens. Germination tests were conducted by incubating the seeds on a moist filter paper in Petri dishes at 15/20 °C with 12 h photoperiod. Protrusion of radicle was observed under a magnifying lens and counted daily for 30 d. Seeds from the impermeable group that remained ungerminated were treated with HCl for 30 min and placed on the moist substrate for an additional 30 days.

### Statistical analysis

Germination percentage of control (untreated) seeds was tested using a two-way ANOVA, with temperature and light conditions as two independent factors. Data were arcsine transformed before analysis to improve normality but actual percentages are reported. Germination percentage of feeding trials was also tested for statistical significance using GLM. We compared the effects of seed egestion time and nature of seeds, i.e. permeable and impermeable. However, unlike the control germination experiment, we fed large number of seeds and were interested in the effect of seed egestion time. For such data, arcsine transformations are not appropriate due to the variation in seed number collected between different periods and normalization of data using the arcsine method assumes that the sample size is the same and places severe limitations at extreme ends of distribution^46^. Consequently, we used log-transformation to improve normality. Multiple means were compared using LSD *post-hoc* test to determine the level of significance (*P* < 0.05). All the analyses were performed in SPSS. Ver. 21.

## Results

### Seed weight and moisture content

The average weight of 100 seeds was 0.2118 ± 0.0113 g. Average moisture content of the seeds at time of collection was 9.78 ± 0.23%.

### Imbibition test

**Figure 2:**
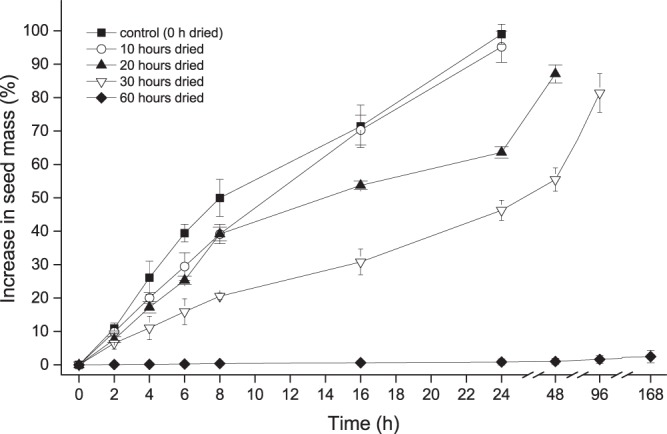
Percentage increase in seed mass when placed on a moist environment after being dried to different periods above silica gel. Error bars represent the standard deviation.

**Figure 3:**
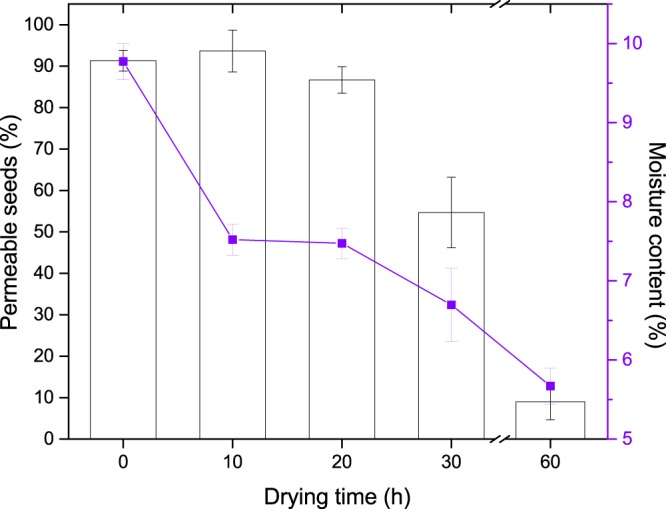
Relationship between moisture content and proportion of permeable seeds in *Astragalus adsurgens*.

The mass of fresh seeds increased rapidly when placed in a moist environment by 50 ± 5.53% in 8 h and 99 ± 2.97 in 24 h (Fig. 2). Many seeds began to show visible germination between 20 and 24 h. Seeds dried above silica gel continued to lose moisture. Fresh seeds with moisture content of 9.78% declined to 7.5% within 10 h of drying, but moisture loss after this was comparatively slower, and reached 5.7% after 60 h. An imbibition test after different periods of drying showed that seeds became impermeable after 60 h, with 87% of the seeds impermeable to water (Fig. 3).

### Seed germination

**Figure 4:**
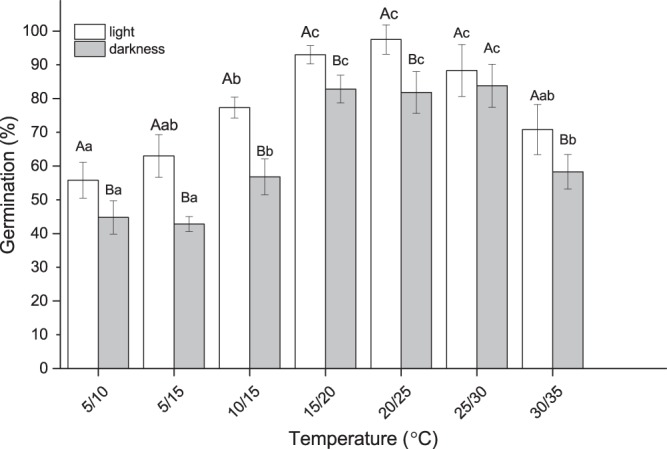
Germination percentage of freshly collected *Astragalus adsurgens* seeds at different temperatures in light and darkness. Error bars represent the standard deviation. Different upper-case letters indicate significant difference between groups under light and darkness. Different lower-case letters denote significant differences within group at different temperatures.

Fresh seeds germinated at a wide-range of alternating temperatures between 5 and 35 °C in both light and darkness (Fig. 4). In seeds incubated at low temperatures between 5 and 10 °C, germination percentage was low (Fig. 4; *P* < 0.05) and seeds took almost 9 d to complete germination (time for 50% germination; t_50_ = 6 days), after which no additional seeds germinated until 30 days. When incubated at a range of temperatures between 15 and 30 °C, germination percentage was significantly higher irrespective of light, and first visible germination was observed within 10 h (t_50_ = 3 days). At higher temperatures above 30 °C, germination was low and most of the seeds that germinated did so within first three days (t_50_ = 2 days) and no additional germination was recorded after three days. There was a higher percentage germination of seeds incubated at temperatures between 15 and 30 °C (Fig. 4; *P* > 0.05). The optimal temperature for germination was 15/20 °C with a 12 h photo period.

### Effect of HCl on dormancy break

**Figure 5:**
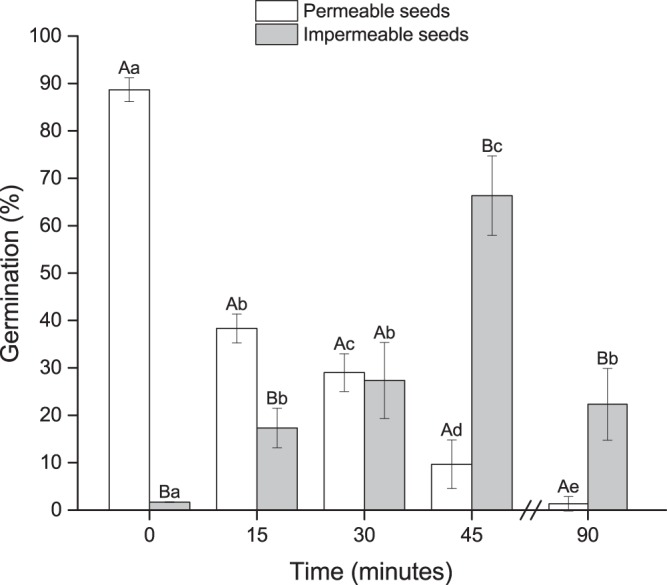
Effect of HCl on germination of permeable and impermeable *Astragalus adsurgens* seeds. Error bars represent the standard deviation. Different upper-case letters indicate significant differences between permeable and impermeable seeds. Different lower-case letters indicate significant differences within group for different treatment periods.

Seeds with permeable and impermeable coats responded significantly different to HCl treatment (*P* < 0.05). There was a significant effect of duration in acid environment and final germination (*P* < 0.05). For permeable seeds, an increased duration in acid environment significantly decreased the germination percentage (Fig. 5). However, impermeable seeds increased germination compared with control after 15, 30 and 45 min, but 90 min in an acid environment significantly reduced the germination percentage compared with 45 min (Fig. 5).

### Seed coat features

Electron micrographs showed morphological and anatomical features similar to seed coats of the subfamily Papilionoideae with four distinctive structures: (1) hilum; (2) micropyle, lies on the side of hilum but appeared to be covered by hilum; (3) lens located on the side of hilum; and (4) extra-hilar region; which is the whole seed coat without hilum, micropyle and lens.

**Figure 6:**
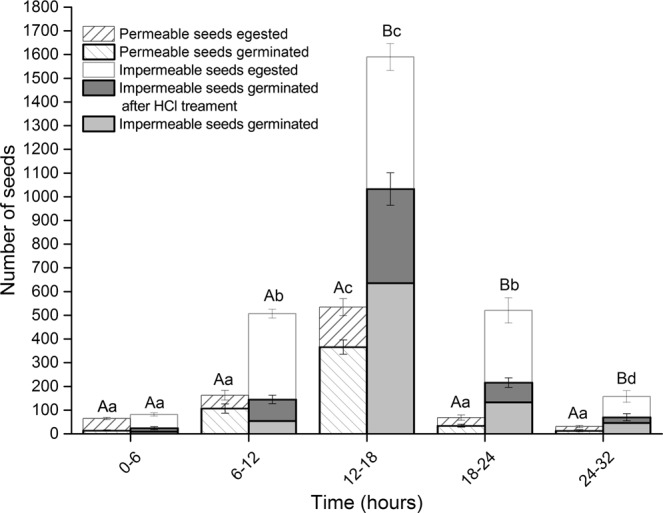
Electron micrographs of (**a**) untreated impermeable seed; (**b**) whole view of impermeable seed treated in HCl for 15 minutes; (**c**) close-up of lens region in 15 minutes HCl treated (d) 30 minutes HCl treated (**e**) lens region in 45 minutes HCl treated; (**f**) whole seed view after 45 minutes HCl treated; (**g**) close-up of cracks in 45 minutes HCl treated; (**h**) whole seed view after 90 minutes HCl treated; (**i**) whole seed view after 90 minutes HCl treated showing smaller cracks; (**j**–**l**) completed damaged coats after 90 minutes HCl treated *Astragalus adsurgens* seeds. H, hilum; M, micropyle; HS, Hilar slit; L, lens; C, cracks.

To further understand the effects of HCl, eight impermeable seeds each were treated in HCl for 15, 30 and 45 minutes. These seeds were then scanned under SEM and the results are presented in Table 1. Impermeable seeds treated with HCl resulted in a narrow linear opening of the hilum (Fig. 6). The lens and extra-hilar region remained intact if the seeds were placed in HCl for only 15 minutes. However, longer exposure to HCl resulted in opening of lens and extra-hilar region (Table 1). Interestingly, some seeds coats of the extra-hilar region also cracked, but predominantly in seeds treated with HCl for 90 min (Table 1; Fig. 6).

**Table 1:** Structural changes in dormancy broken *Astragalus adsurgens* seeds examined under scanning electron microscope after different durations in HCl.

Treatment	Number of seeds scanned	Number of seeds with hilar slit cracked	Number of seeds with lens cracked	Number of seeds with extra-hilar region cracked
HCl for 15 min	8	5	0	0
HCl for 30 min	8	8	0	2
HCl for 45 min	8	7	2	3
HCl for 90 min	8	8	3	7

### Seed feeding trials

**Figure 7:**
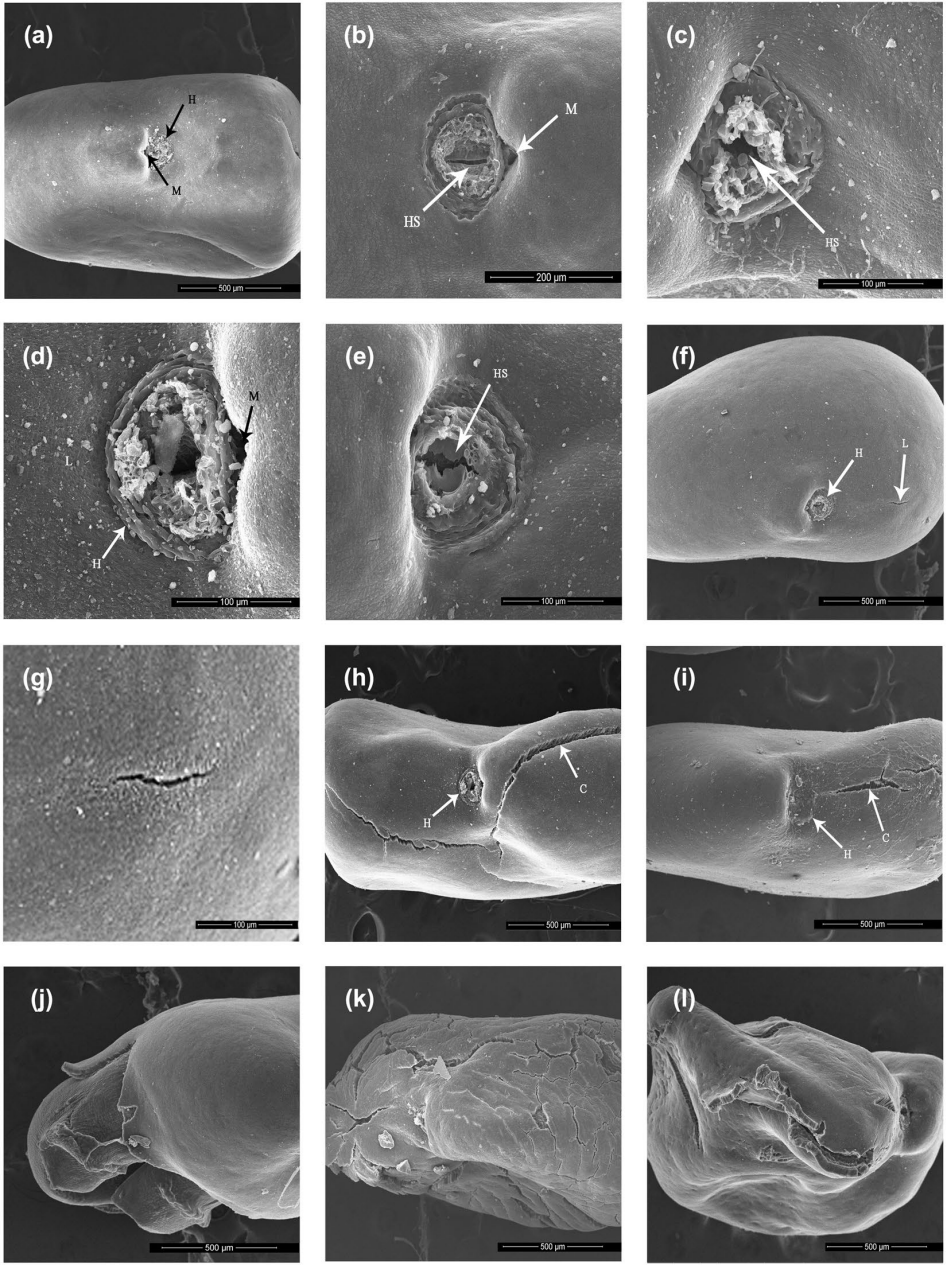
Effect of gut passage on permeable and impermeable seed germination. Seeds recovered were grouped based on time. Different lower-case letters indicate significant differences within group at different time. Different upper-case letters indicate significant differences between permeable and impermeable seeds.

Overall, significantly less permeable seeds were recovered in dung than impermeable seeds (*P* > 0.05; Fig. 7). There was a significant effect of seed coat type (whether permeable or impermeable) on germination rate (*P* < 0.05). Five hundred and thirty-four permeable seeds germinated successfully out of 5400 seeds fed in three replicates of 1800 seeds (Fig. 7). In contrast, 879of the 5400 impermeable seeds egested were able to germinate successfully (Fig. 7). An additional 617 seeds germinated when treated with HCl for 30 min (Fig. 7). Dissection revealed that internal structures of the ungerminated seeds were damaged. Egestion time also had a significant effect on germination percentage (*P* < 0.05). Germination rates were extremely low in seeds egested within the first 6 h and after 24 h (Fig. 7). Twenty-eight impermeable seeds were recovered from dung after 32 h, but none of them imbibed or germinated. Irrespective of seed coat type, seed recovery was high between 12 and 24 h (Fig. 7).

## Discussion

Naturally dispersed *A*. *adsurgens* seeds imbibed water and did not exhibit physical dormancy (Figs 2 and 4). In many non-dormant desert plants, seed germination is controlled principally by temperature and light^47^. Here, *A*. *adsurgens* seeds germinated at a wide-range of temperatures both in light and darkness (Fig. 3); consistent with seeds from the Qubei deserts of China^5^. Three different trends in germination were observed based on the temperature and light conditions: low germination at temperatures below 10 and above 30 °C, whereas higher germination was observed in temperature between 15 and 30 °C.

Maternal environment plays an important role in drying the seeds^17^. In Inner Mongolian grasslands, seeds of *A*. *adsurgens* undergo maturation drying from July to August, when both humidity and rainfall are high (Fig. 1). Such conditions do not favor the drying of the seeds. Accordingly, freshly collected seeds were moist and permeable to water. However, seeds might dry below the threshold required for the induction of coat impermeability with a warmer climate resulting from climate change, abnormally hot summers, or drought. Our own preliminary studies from particularly dry years have indicated that plants could produce seeds with impermeable coats. Further, our results imply that permeable seeds of *A*. *adsurgens* can become impermeable in the soil whilst waiting for germination cues (i.e. secondary dormancy). Thus by producing a cohort of permeable and impermeable seeds at different years the species might increase their ability to persist in soil and colonize at a latter year. Our results here demonstrate empirically that drying seeds induces impermeability (Fig. 3). Seeds dried for 10 hours were predominantly permeable and absorbed water similarly to control seeds (see Figs 2 and 3). However, further drying increased the proportion of impermeable seeds. When dried for 60 h, 87% of seeds became impermeable (Fig. 3) and water uptake in these seeds was extremely low, even after three weeks.

Our results suggest the threshold moisture content required for the onset of impermeability in *A*. *adsurgens* is approximately 6.5%. This value is significantly lower than those of most other species studied to date^10^. Although, impermeability is induced at much low moisture content in some other species (e.g. 4% in *Cajanus cajan*^48^), the lower moisture threshold in *A*. *adsurgens* might be an adaptation to increase the proportion of permeable seeds in temperate locales, where dormancy-breaking conditions are different to those experienced in the tropics. In temperate environments, temperatures often fall below 0 °C and freeze-thaw cycles can make *A*. *adsurgens* seeds permeable^2^; although most overwintering seeds are held in stasis under snow cover. Alternatively, the difference in moisture content required for the onset of impermeability could be attributed to reasons relating to phylogeny, seed size, seed-to-seed variation, or age of the mother plant. The effect such factors have on the species-specific moisture thresholds requires further study.

Many *Astragalus* species produce seeds with impermeable coats^49–56^. Contrasting results regarding seed permeability in *A*. *adusurgens* might result from the storage conditions employed before experimentation. Tetsuya and Sayaka^2^ stored seeds in air-tight containers containing silica gel for 6 d at 5 °C in darkness. Our results showed that drying seeds above silica gel for 60 h is sufficient to render them impermeable. To confirm this, we dried fresh seeds for 6 d at 5 °C in darkness and found that 93% of seeds became impermeable. This suggests the physiological maturity of seeds tested by Tetsuya and Sayaka^2^ was altered. In contrast, Tobe and Gao^5^ stored seeds at room temperature for 16 months and then at 0 °C for 2–4 months before germinating them. Under these conditions seeds might have maintained a higher moisture content and not developed impermeability. Consequently, a higher number of seeds germinated at different temperatures.

In many Fabaceae species, the lens or pseudo-lens acts as the primary water-gap through which water enters. However, imbibition can occur elsewhere in some species (see Introduction). For instance, imbibition of many Australian Fabaceae species occurs at the hilum and/or micropyle^33^. In some species, covering the lens with petroleum jelly did not prevent germination. Egley^10^ reported similar results in *Crotalaria spectabilis*. Furthermore, in some Papilionoideae species, the ‘hilar slit’ is the primary water entry point, with the lens acts as a secondary water gap^38^. In contrast to these findings, HCl treatment of *A*. *adsurgens* seeds made the hilar region crack in a narrow, linear shape and the lens opened in few seeds (Table 1), suggesting that the hilar region could serve as a water entry site. This result, however, contradicts studies of *Astragalus cicer*, that opened the lens following treatment with sulfuric acid treatment^53^. Nevertheless, our results are consistent with Hu *et al*.^57^, who showed that *Vigna oblongiflolia* seeds treated with hot-water and sulfuric acid became permeable through cracks in the hilum and extra-hilar region. Likewise, Hu *et al*.^57^ revealed that *S*. *alopecuroides* seeds dipped in hot-water cracked first at the hilum and later at the lens. In the present study, we found that *A*. *adsurgens* seeds treated with HCl for 15 and 30 min formed cracks on the hilar region, whereas HCl treatment of 45 and 90 min resulted in wider cracks on the hilar and extra-hilar regions (Fig. 6). Tetsuya and Sayaka^2^ similarly reported that *A*. *adsurgens* seeds treated with sulphuric acid had cracks on the seed coat.

*A*. *adsurgens* are often consumed by animals. Herbivores offer dual benefits for PY species, namely, dispersal to new environments and making seed coats permeable in their gut. Here, germination rates were improved by gut passage, suggesting dormancy is broken by gut passage; presumably by acid scarification. Acidic environments can damage seeds with soft coats and thereby lower germination rates. Indeed, permeable seeds treated with HCl exhibited lower germination rates when compared to impermeable seeds (Fig. 5). Irrespective of the seed coat, most seeds were egested between 12 and 24 h (Fig. 7), potentially indicating an ‘optimal time’ for dormancy to break. Examples of such trends are available in the literature^58^, though this ‘optimal time’ might vary between species and is principally affected by seed size and herbivore type, among other factors [see^39^]. Small seeds are known to pass quickly through the gut compared with large seeds^59,60^. Given the size of a cow’s intestine, it might be argued that *A*. *adsurgens* seeds are small and are thereby passed rapidly, minimizing damage to permeable seeds both. Nevertheless, gut passage enabled impermeable seeds to break dormancy.

In summary, we have shown that reduction of seed moisture content facilitates the development of impermeable coats in *A*. *adsurgens*. Physical dormancy is broken by treatment with HCl and after gut passage. Water imbibition occurs at the hilum or extra-hilar region, resulting in germination. This is confusing, given the lens typically plays a primary or secondary role in water uptake of Papilionoideae species. However, dormancy break by other means, particularly ecologically meaningful seasonal temperature changes, must be investigated to better understand the role of the lens in controlling water entry.
